# Five cuts from herring (*Clupea harengus*): Comparison of nutritional and chemical composition between co-product fractions and fillets

**DOI:** 10.1016/j.fochx.2022.100488

**Published:** 2022-10-25

**Authors:** Haizhou Wu, Bita Forghani, Mehdi Abdollahi, Ingrid Undeland

**Affiliations:** Department of Biology and Biological Engineering-Food and Nutrition Science, Chalmers University of Technology, SE 412 96 Gothenburg, Sweden

**Keywords:** *Clupea harengus*, Fish by-products, side streams, Fatty acids, Amino acids, Minerals, Vitamins

## Abstract

Weight distribution, proximate composition, fatty acids, amino acids, minerals and vitamins were investigated in five sorted cuts (head, backbone, viscera + belly flap, tail, fillet) emerging during filleting of spring and fall herring (*Clupea harengus*). The herring co-product cuts constituted ∼ 60 % of the whole herring weight, with backbone and head dominating. Substantial amounts of lipids (5.8–17.6 % wet weight, ww) and proteins (12.8–19.2 % ww) were identified in the co-products, the former being higher in fall than in spring samples. Co-product cuts contained up to 43.1 % long-chain n-3 polyunsaturated fatty acids (LC n-3 PUFA) of total FA, absolute levels peaking in viscera + belly flap. All cuts contained high levels of essential amino acids (up to 43.3 %), nutritional minerals (e.g., iodine, selenium, calcium, and iron/heme–iron), and vitamins E, D, and B12. Co-products were, in many cases, more nutrient-rich than the fillet and could be excellent sources for both (functional) food and nutraceuticals.

## Highlights

• Head, backbone, viscera + belly flap, and tail made up ∼ 60 % of the whole herring weight.
• All cuts were rich protein (up to 19.2% ww) with balanced essential amino acids.
• Substantial amounts of LC n-3 PUFAs & LC MUFAs were identified in the co-products..
• All cuts were rich in Vit E, D, and B12, as well as I, Se, Ca, and heme-Fe.
• The co-products did not pose any risk to human health based on Hg, Pb, and Cd.

## Graphical abstract

**Figure:**
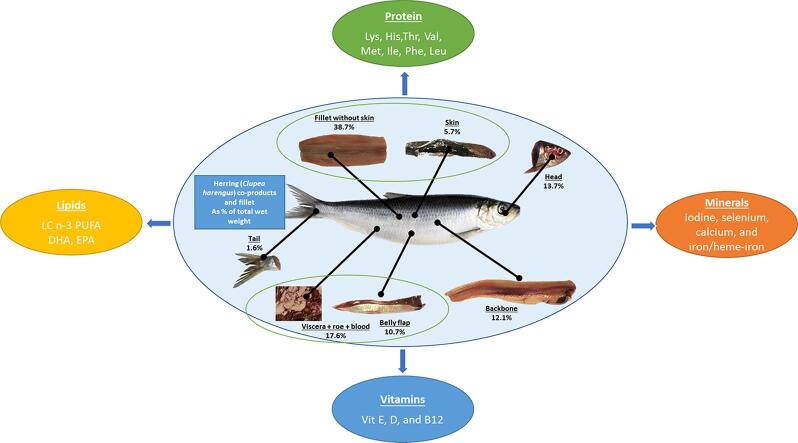

## Introduction

Fish and fish products contain high-quality proteins, long-chain n-3 polyunsaturated fatty acids (LC n-3 PUFA), and important micronutrients, such as vitamins and minerals (Bazarnova et al., 2020, USDA, 2019). Consequently, the Food and Agriculture Organization (FAO) of the United Nations reported that the apparent global fish consumption over the past 60 years had increased significantly more than the world population growth (FAO, 2020). However, the currently available fish resources and the expected aquaculture expansions cannot cover the increased global demand based on the endurance of ecosystems (FAO, 2020). Therefore, this demand has increased interest in optimizing the utilization of caught or harvested fish for food.

In the fishing industry, about 70 % of the total landings of fish are processed into fillets, an operation which results in 20–80 % (w/w) of fish co-products (Ghaly et al., 2013). Utilizing these co-products for food rather than feed or other lower-value applications would be a promising way to meet the increasing demand for fish, fish products, or ingredients. To date, many different routes have been explored for the valorization of non-fillet parts into products such as minces, protein isolates, protein hydrolysates, and oils (Nikoo et al., 2019, Sajib et al., 2022, Wu et al., 2021). However, the suitability of different co-products cuts for these additional options differs. For example, the fish liver is traditionally used for oil production (Rustad, Storrø, & Slizyte, 2011), while backbones are reported as highly suitable for mince and protein isolate production (Wu, Abdollahi, & Undeland, 2021).

Systematic data on fish co-product chemical and nutritional composition is a prerequisite for tailoring their utilization for different types of products or ingredients. However, data are still limited to relatively few species, and most studies have primarily focused on lipids. Ahmmed et al. (2021) investigated the lipidomic profile (phospholipids, triglycerides, fatty acid profile, positional distribution of fatty acids) of blue mackerel (*Scomber australasicus*) co-product cuts (head, skin, roe, and male gonad). This investigation revealed considerable n-3 PUFA and phospholipids levels in all four cuts, especially in roe. Additionally, Abiona et al. (2021) investigated the characteristics of oil extracted from the head and gills of catfish (*Claris macrocephalus*) as well as Atlantic mackerel (*Scomber scombrus*). Notably, they found that it was enriched in n-3 PUFA, such as eicosapentaenoic acid (EPA) and docosahexaenoic acid (DHA). Munekata et al. (2020) performed a nutritional characterization of sea bass (*Dicentrarchus labrax*) co-product cuts (skin, guts, gills, liver, head, and fishbone), in which they suggested using the guts and liver to obtain monounsaturated fatty acids (MUFA) and LC n-3 PUFA. Likewise, they proposed using the heads, bones, and gills to recover essential minerals, such as calcium, phosphorus, and manganese, and using the skin to obtain proteins and essential amino acids (EAA), such as lysine and leucine. Furthermore, recent literature also reports on the nutritional profiles of individual co-product parts from gilthead sea bream (*Sparus aurata*), and meagre (*Argyrosomus regius*) (Kandyliari et al., 2020), Black Sea anchovy (*Engraulis encrasicholus*) (Gencbay & Turhan, 2016) and gilthead sea bream (*Sparus aurata*) (Pateiro et al., 2020).

In previous studies, it has been reported that gutted herring (*Clupea harengus)* and herring fillets are rich in high-quality proteins with a balanced EAA profile, vitamins B12, vitamin D, minerals (specifically calcium, potassium, magnesium, and iodine), and LC n-3 PUFA (EPA, DHA, and docosapentaenoic acid [DPA]) (Bazarnova et al., 2020, Hamre et al., 2003, Marmon and Undeland, 2010). However, no systematic mapping of nutrients in different cuts of herring has been reported, including the parts making up the co-product fraction. This could originate from herring co-products commonly mixed in one container, without any sorting, to be used in animal feed and oil production. Large parts of the herring landings also go directly to such production, without taking out the fillet. Based on the FAO (2020), herring is captured globally at an average of 2,162 thousand tonnes per year and is ranked the 4th most landed species between 1950 and 2017. It has been identified as one of the most climate-friendly fish species yielding only 0.7 kg of CO_2_ equivalents per kilogram (Bianchi et al., 2022). Furthermore, most stocks are still considered sustainable (FAO, 2020). Therefore, more profound knowledge about the distribution of macro- and micronutrients among the different co-product cuts could contribute to a changed utilization pattern of this species, with more going to food and less to feed.

The present study investigated weight distribution, crude composition, and nutrient profile (e.g., fatty acids, amino acids, minerals, and vitamins) of sorted herring filleting cuts, including the head, backbone, viscera + belly flap, tail, and fillet. Furthermore, differences between typical catches from the spring and fall seasons regarding these parameters were also analyzed.

## Materials and methods

### Preparation of herring co-products and fillets

**Fig. 1:**
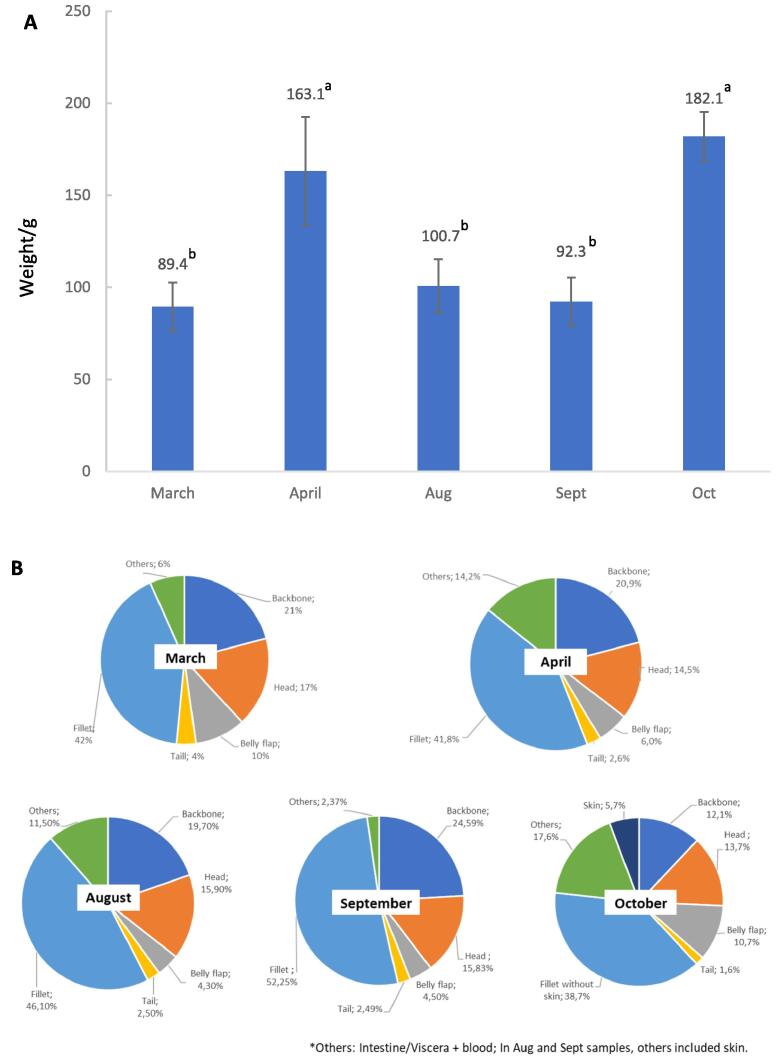
Average weight (A) of the whole herring used in the five different co-product samplings (n = 10) and the weight distribution (B) of the sorted cuts from each month (n = 15).

Five batches of herring caught in 2020 (March, April, August, September, and October) were filleted and sorted by a filleting machine (Model Baader 36, Nordisher Maschinenbau Rudolf Baader Gmbh, Lubeck, Germany) at Sweden Pelagic AB (Ellös, Sweden). The geographic origin and pre-processing storage time are shown in Table S1. Five sorted cuts (head, backbone, viscera + belly flap, tail, and fillet) were collected, covered with ice-filled plastic bags, and transported within 3 h to the marine lab at Chalmers University of Technology. Upon arrival, all cuts and whole herrings were photographed and weighed (Figs. 1 and S1). Skin-on fillets were produced for samplings from March, April, and October, while fillet samplings from August and September were de-skinned. In the October sampling, we decided to weigh the skin separately why it was manually removed from the skin-on fillets obtained prior to weighing. In parallel, the sorted herring cuts were ground using a table-top meat grinder (C/E22 N, Minerva Omega Group, Italy) equipped with a plate with 4.5 mm holes and subsequently pooled and mixed to complete homogeneity. Samples were packed in Polynova plastic bags (89 mm × 114 mm, 50 my), and each bag contained 80–90 g of the sample. Any air in the bag was manually squeezed out, and samples were stored at −80 °C until subsequent analysis. Part of the samples from each cut was dried by freeze-drying (at − 53 °C and<0.01 hPa pressure for 24 h) using a freeze dryer (Heto LyoPro 3000, Heto/Holten A & S, Allerød, Denmark) and then ground to a fine powder (Wu, Forghani, Abdollahi, & Undeland, 2022). Finally, these freeze-dried samples were used to analyze amino acid profiles, minerals, and vitamins D and B12.

### Proximate composition

The proximate composition of all cuts from two of the catching occasions (April and October) was measured according to the methods described by Wu et al., 2022, Abdollahi et al., 2021. In brief, moisture content was measured gravimetrically by overnight heating at 105 °C. The ash content was also analyzed gravimetrically by heating the samples at 550 °C in a furnace for 6 h. Total nitrogen content was detected using a LECO nitrogen analyzer (Supplementary Table S2). To analyze crude lipids, 2.0 g of herring mince were mixed with 20 mL of chloroform/methanol (2:1). The mixture was homogenized using a polytron (T18 digital Ultra-Turrax, IKA, Germany) for 15 s at 12,000 rpm, and 6.16 mL of 0.5 % NaCl was added to the samples. After adding NaCl, samples were vortexed for 30 s, followed by centrifugation at 2,000 × g for 10 min. After centrifugation, 4 mL of lower phase (chloroform) were transferred into a glass tube and then evaporated until dry under oxygen-free nitrogen gas at room temperature, followed by weighing the lipid fraction for crude lipid analysis (Wu et al., 2022). Another 4 mL of the chloroform phase was transferred into a new glass tube with Teflon screw-caps and was stored at −80 °C for subsequent analysis of fatty acid profiles or vitamin E content.

### Fatty acid profile

The fatty acid composition of the lipid fraction from section 2.2 was measured according to the method described by Wu et al. (2022). Briefly, heptadecanoic acid (C17:0) (Larodan AB 10–1700-13) was added to all samples as an internal standard prior to methylation. The samples were then methylated using the in-house methanolic-HCl transesterification method as described by Cavonius et al. (2014) (Supplementary Table S2). Following methylation, the samples were subjected to gas chromatography-mass spectrometry (GC–MS) analysis using an Agilent 7890 A GC system (Agilent Technologies, Santa Clara, CA, USA) equipped with a J&W DB-wax column (30 m × 0.25 mm × 0.25 μm) and interfaced with an Agilent 5975C triple-axis mass spectrometric (MS) detector. The standard GLC 463 (Nu-Chek prep, Inc., Elysian, USA), containing 52 different fatty acid methyl esters (FAMEs), was used to identify the different peaks in our herring samples.

### Amino acid profile

The amino acid composition of the freeze-dried samples from section 2.1 was detected following the methods detailed in Özcan and Şenyuva, 2006, Abdollahi et al., 2021. In brief, 100 mg of freeze-dried powder was transferred into screw cap glass tubes, and 4 mL H_2_O and 4 mL pure HCl were added to the tubes. The glass tubes were then heated for 24 h at 110 °C. After heating, 8 mL of the sample was moved from a glass tube to a 15 mL plastic tube. The glass tube was then rinsed with 2 mL of distilled H_2_O to bring the total sample volume to 10 mL, and 1 mL of each diluted sample was transferred to a 1.5 mL Eppendorf tube and centrifuged for 3 mins at 16,000×g. The supernatant was further diluted 20 times with 0.1 N HCl, loaded into glass vials, and automatically analyzed using LC-MS (Supplementary Table S2). Thermo Scientific Pierce Amino Acid Standard (NCI0180. 20088) with a quantitative mixture of 18 amino acids was used as an external standard to prepare the standard curve.

### Minerals

The freeze-dried samples from section 2.1 were subjected to acidic microwave digestion based on the methods reported by Larsson, Almgren, and Undeland (2007). Following digestion, the samples were appropriately diluted and used to measure Na, K, Ca, Mg, Fe, Zn, Cu, I, Se, As, Cr, Hg, Pb, Cd by inductively coupled plasma mass spectrometry (ICP-MS, see Supplementary Table S2) (Wu et al., 2022).

### Heme-iron analysis

Total heme was detected using the acetone-based method described in our recent study (Wu, Ghirmai, & Undeland, 2020). Bovine hemoglobin (0.1, 0.5, 1, 5, and 10 μM) was used to prepare a standard curve, which we used to calculate the total heme in the herring samples. The heme–iron content was then calculated using the factor of 0.0882 μg iron/μg heme (Wu, Abdollahi, & Undeland, 2021).

### Analysis of vitamin E, D, and B12

The content of vitamin E (α-tocopherol) in lipid fraction samples from section 2.2 was analyzed using high-performance liquid chromatography (HPLC) with fluorescence detection according to the method described by Wu et al. (2022). Quantitative analysis of tocopherols was performed using calibration curves of α-, β-, γ- and δ-tocopherols (Sigma-Aldrich, Steinheim, Germany). The freeze-dried samples from section 2.1 were used to analyze the overall vitamin D and B12 content. In short, the measurement of vitamin D3 involved saponification, extraction, purification by solid-phase extraction (SPE), and quantification by reverse-phase HPLC using internal standards (Alfa Aesar B22524) (Abdollahi, Wu, & Undeland, 2021). A microbiological assay determined vitamin B12 in an accredited laboratory (National Food Agency of Sweden), and was performed using the microbiological assay and turbidimetric detection of the growth of *Lactobacillus casei, subsp. Rahmnosus* (L. rahmnosus, equivalent to L. casei American Type Culture Collection, ATCC 7469) (AACC, 2000).

### Statistical analysis

All statistical analyses were conducted using SPSS software (IBM SPSS Statistics Version 22, IBM Inc., Chicago, USA). The results were reported as mean ± standard deviation (SD) (n ≥ 2). Duncan’s multiple range test was used to compare the means. Variance (ANOVA) was used to analyze the significant differences between different cuts and seasons (spring, represented by April; fall, represented by October). The threshold for significance for all tests was set at p < 0.05.

## Results

### Weight distribution of whole herring and co-product cuts

Whole herring and their cuts were sampled from five different catches in 2020 (March, April, August, September, and October). Table S1 shows the five samplings' geographic origin and pre-processing storage time. The catches used in this study represent typical herring landings processed at Sweden Pelagic AB in Ellös, Sweden’s largest herring processor. Fig. 1A and Fig. S1 show the average weight and size of herring from the five catches used. As observed, the weight of the herring ranged from 89.4 g to 182.1 g, and the length from 21.5 to 27.5 cm. The April and October samplings were significantly (p < 0.05) larger than those from March, August, and September (Fig. 1A). Importantly, April and October samplings were selected beforehand for thorough investigations of nutritional composition, given that April represents the middle of the spawning period for spring spawning herring, and October represents the mid-fall season related to catching and processing herring in Sweden. Fig. S2 shows photo documentation of the cuts collected from all five months' samplings: backbones, heads, viscera, belly flap, and tail. The sizes of the cuts were documented and reflected the size of the fish. An unexpected observation was that the April and October samplings comprised herrings with large amounts of liver, roe, and milt, consequently making the viscera + belly flap cut larger. Notably, the herring included in this study comprised both spring and autumn spawners which could be explained by the large geographical spread (North Sea, Kattegat, and the Limfjord).

Fig. 1B shows the weight distribution of the sorted cuts from all five months' samplings. In the viscera + belly flap cut, all viscera were mixed and contained a lot of blood and other liquids, so it was difficult to pick them out to weigh on a one-by-one basis. The weight of the viscera was therefore included in the “others” cut together with blood, for example. Specifically, it was calculated by subtracting the average weight of the tail, belly flap, head, backbone, and fillet from the average weight of the whole herring. Among the herring co-products, backbones and heads were the largest cuts (12.1–24.6 % and 13.7–17 % of the entire herring weight, respectively). This was followed by the cut denoted as “others” (2.4–17.6 %), which was combined with the belly flap during sorting (4.5–10.7 %). The smallest cut reported was the tail (1.6–4 %). The cut called “others” varied extensively due to the presence or absence of roe/milt. The fact that herring consume more in fall than in spring may explain why the cut called “others” was higher in fall than in spring. Based on manual skin removal in the October trial, it was found that the skin contributed to about 5 % of the total fish weight. The fact that the fillet only contributed to 42–45 % of the whole fish weight highlights the importance of adding value to the co-products that can be processed from the fish. Based on its visual appearance, the backbone cut was identified as particularly relevant to valorize since it contains a significant amount of residual muscle and appears similar to that of a small fillet.

### Proximate composition

**Fig. 2:**
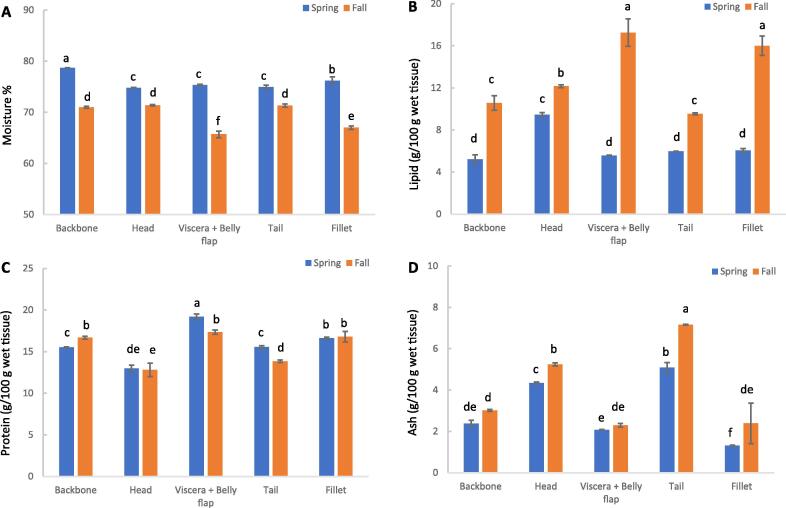
Moisture (A), lipid (B), protein (C), and ash (D) contents of sorted herring filleting co-products and fillets from spring (April) and fall (October).

Fig. 2 shows the proximate composition of the herring filleting co-products (backbone, head, viscera + belly flap, tail) and the fillet collected in April and October. Data are expressed on a wet weight (ww) basis, regarded as most relevant for fish processors. Moisture was significantly (p < 0.05) higher in the spring than in fall samples from all five investigated cuts (Fig. 2A). Conversely, total lipid content was substantially higher in fall samples than spring samples (Fig. 2B). Aligned with these results, Fig. 2B shows that the viscera + belly flap and fillet were the co-product cuts having the highest lipid content in the October sampling, followed by head > backbone ≈ tail. In April, on the other hand, the head had the highest lipid content, followed by all the other parts, which could not be significantly differentiated. Notably, a substantial amount of protein (12.8–19.2 %) was found in all cuts from both seasons (Fig. 2C). The protein contents in the head, fillet, and backbone were similar in spring and fall samples. However, viscera + belly flap and tail from spring showed a significantly (p < 0.05) higher protein content than corresponding fall samples. Fig. 2D shows that compared to other cuts, the tail and head had the highest ash content, up to 7.2 % and 5.2 %, respectively. Furthermore, excluding viscera + belly flap, all other four cuts showed a significantly higher ash content in fall than in spring (p < 0.05 for all groups).

### Fatty acid profile

Table 1 shows the absolute content (g/100 g ww) of individual fatty acids, total saturated fatty acids (SFA), MUFA, LC MUFA, PUFA, and LC n-3 PUFA in the different cuts as well as their percent of total fatty acids. In general, seven fatty acids (C14:0, C16:0, C16:1, C18:1, C22:1, C20:5, and C22:6) dominated among the identified fatty acids (>5%), regardless of cuts and season. The absolute fatty acid content was significantly different across the different cuts (Table 1). For example, the head contained the highest SFAs (5.7 g/100 g ww), followed by viscera + belly flap ≈ tail > fillet > backbone in fall samples. The MUFA content was also higher in the head and viscera + belly flap than in the other cuts. Regarding the LC MUFA, the head and viscera + belly flap had the highest level (2.9 g/100 g ww), followed by tail > fillet ≈ backbone. Furthermore, the PUFA and LC n-3 PUFA contents were highest in viscera + belly flap (up to 7.1 and 6.5 g/100 g ww, respectivey), followed by fillet > backbone > tail ≈ head.

**Table 1:** Content of fatty acids in g/100 g wet tissue and as % of total fatty acids of sorted herring filleting co-products and fillet from spring (April) and fall (October).

Fatty acids	Backbone		Head		Viscera + Belly flap		Tail		Fillet	
	Spring	Fall	Spring	Fall	Spring	Fall	Spring	Fall	Spring	Fall
C12:0	0.005 ± 0.0002 (0.12 %)	0.009 ± 0.0007 (0.12 %)	0.017 ± 0.0005 (0.21 %)	0.020 ± 0.002 (0.19 %)	0.002 ± 0.0001 (0.05 %)	0.013 ± 0.0014 (0.09 %)	0.005 ± 0.0007 (0.09 %)	0.015 ± 0.0011 (0.15 %)	0.004 ± 0.0001 (0.08 %)	0.010 ± 0.0005 (0.10 %)
C14:0	0.389 ± 0.0248 (9.31 %)	0.678 ± 0.0274 (9.28 %)	1.351 ± 0.0873 (16.33 %)	1.615 ± 0.1566 (15.42 %)	0.225 ± 0.001 (5.52 %)	1.189 ± 0.1014 (7.66 %)	0.389 ± 0.108 (7.63 %)	1.301 ± 0.1003 (13.60 %)	0.351 ± 0.0172 (7.74 %)	0.856 ± 0.121 (8.41 %)
C16:0	0.886 ± 0.0406 (21.21 %)	1.893 ± 0.0406 (25.56 %)	2.661 ± 0.1468 (32.16 %)	3.512 ± 0.1829 (33.52 %)	1.029 ± 0.0204 (25.24 %)	3.8 ± 0.2695 (24.50 %)	1.11 ± 0.1659 (21.79 %)	3.121 ± 0.2338 (32.62 %)	0.977 ± 0.037 (21.71 %)	2.537 ± 0.2806 (24.90 %)
C18:0	0.090 ± 0.0048 (2.16 %)	0.175 ± 0.002 (2.35 %)	0.318 ± 0.0072 (3.84 %)	0.407 ± 0.0122 (3.89 %)	0.082 ± 0.0012 (2.02 %)	0.345 ± 0.0275 (2.22 %)	0.110 ± 0.0166 (2.16 %)	0.316 ± 0.0213 (3.31 %)	0.079 ± 0.0035 (1.76 %)	0.187 ± 0.0241 (1.84 %)
C20:0	0.049 ± 0.0011 (1.18 %)	0.057 ± 0.0012 (0.77 %)	0.106 ± 0.1498 (1.28 %)	0.157 ± 0.0133 (1.50 %)	0.028 ± 0.0006 (0.70 %)	0.089 ± 0.0135 (0.58 %)	0.071 ± 0.0301 (1.40 %)	0.155 ± 0.0056 (1.62 %)	0.058 ± 0.0029 (1.27 %)	0.066 ± 0.0004 (0.65 %)

**ΣSFA**	1.420 ± 0.0716^f^ (33.99 %)	2.812 ± 0.0719^e^ (38.09 %)	4.454 ± 0.3915^c^ (53.83 %)	5.710 ± 0.3671^a^ (54.51 %)	1.367 ± 0.0197^f^ (33.52 %)	5.437 ± 0.4133^ab^ (35.05 %)	1.685 ± 0.3212^f^ (33.07 %)	4.908 ± 0.3622^bc^ (51.29 %)	1.469 ± 0.0606^f^ (32.56 %)	3.657 ± 0.4267^d^ (35.89 %)

C16:1 (n-7)	0.215 ± 0.0117 (5.14 %)	0.442 ± 0.0147 (6.02 %)	0.750 ± 0.0325 (9.06 %)	0.986 ± 0.0791 (9.42 %)	0.139 ± 0.0035 (3.40 %)	0.696 ± 0.0419 (4.49 %)	0.218 ± 0.0528 (4.27 %)	0.781 ± 0.0489 (8.16 %)	0.186 ± 0.0062 (4.15 %)	0.513 ± 0.0741 (5.04 %)
C18:1 (n-9)	0.451 ± 0.0423 (10.80 %)	0.654 ± 0.0159 (8.85 %)	1.366 ± 0.1527 (16.51 %)	1.357 ± 0.0865 (12.95 %)	0.348 ± 0.0175 (8.52 %)	1.122 ± 0.0838 (7.23 %)	0.484 ± 0.1544 (9.50 %)	1.068 ± 0.0151 (11.16 %)	0.362 ± 0.0253 (7.86 %)	0.628 ± 0.0735 (6.16 %)
C18:1 (n-7)	0.080 ± 0.0042 (1.92 %)	0.138 ± 0.0039 (1.87 %)	0.289 ± 0.0081 (3.50 %)	0.332 ± 0.0142 (3.17 %)	0.183 ± 0.0043 (4.48 %)	0.542 ± 0.046 (3.49 %)	0.115 ± 0.0095 (2.25 %)	0.286 ± 0.0276 (2.99 %)	0.075 ± 0.0018 (1.69 %)	0.162 ± 0.015 (1.59 %)
C20:1 (n-9)	0.049 ± 0.0011 (1.17 %)	0.058 ± 0.0029 (0.80 %)	0.202 ± 0.0145 (2.44 %)	0.158 ± 0.0117 (1.51 %)	0.029 ± 0.0009 (0.70 %)	0.089 ± 0.0134 (0.58 %)	0.071 ± 0.0301 (1.40 %)	0.155 ± 0.0058 (1.62 %)	0.058 ± 0.0029 (1.27 %)	0.066 ± 0.0004 (0.65 %)
C22:1 (n-11)	0.472 ± 0.02 (11.29 %)	0.04 ± 0.0012 (0.55 %)	0.127 ± 0.0081 (1.53 %)	0.132 ± 0.0129 (1.26 %)	0.115 ± 0.147 (2.82 %)	0.483 ± 0.6096 (3.11 %)	0.561 ± 0.2581 (11.02 %)	0.103 ± 0.006 (1.08 %)	0.463 ± 0.0444 (9.86 %)	0.373 ± 0.4655 (3.66 %)

**ΣMUFA**	1.267 ± 0.0793^bcd^ (30.33 %)	1.333 ± 0.0386^bcd^ (18.10 %)	2.734 ± 0.2159^a^ (33.04 %)	2.966 ± 0.2045^a^ (28.32 %)	0.812 ± 0.1577^d^ (19.93 %)	2.932 ± 0.4246^a^ (18.90 %)	1.449 ± 0.5049^bc^ (28.44 %)	2.393 ± 0.0731^a^ (25.02 %)	1.145 ± 0.0807 ^cd^ (24.83 %)	1.741 ± 0.3025^b^ (17.09 %)

**ΣLC MUFA**	0.521 ± 0.0211^a^ (12.47 %)	0.099 ± 0.004^a^ (1.35 %)	0.328 ± 0.0226^a^ (3.97 %)	0.291 ± 0.0246^a^ (2.77 %)	0.144 ± 0.1479^a^ (3.52 %)	0.572 ± 0.5963^a^ (3.69 %)	0.633 ± 0.2881^a^ (12.42 %)	0.258 ± 0.0118^a^ (2.70 %)	0.521 ± 0.0474^a^ (11.13 %)	0.439 ± 0.4651^a^ (4.31 %)

C18:2 (n-6)	0.068 ± 0.0054 (1.62 %)	0.119 ± 0.0041 (1.62 %)	0.165 ± 0.0069 (2.00 %)	0.195 ± 0.0114 (1.86 %)	0.059 ± 0.0006 (1.44 %)	0.237 ± 0.0171 (1.53 %)	0.086 ± 0.0197 (1.69 %)	0.200 ± 0.0147 (2.09 %)	0.076 ± 0.0023 (1.70 %)	0.164 ± 0.019 (1.61 %)
C18:3 (n-3)	0.004 ± 0.0006 (0.10 %)	0.010 ± 0.0001 (0.13 %)	0.009 ± 0.0003 (0.11 %)	0.014 ± 0.0001 (0.13 %)	0.008 ± 0.0001 (0.20 %)	0.020 ± 0.001 (0.13 %)	0.009 ± 0.0056 (0.18 %)	0.013 ± 0.0001 (0.14 %)	0.010 ± 0.0031 (0.19 %)	0.012 ± 0.0021 (0.12 %)
C18:4 (n-3)	0.043 ± 0.0038 (1.04 %)	0.092 ± 0.0037 (1.26 %)	0.096 ± 0.0045 (1.16 %)	0.128 ± 0.0029 (1.23 %)	0.034 ± 0.0003 (0.83 %)	0.183 ± 0.013 (1.18 %)	0.049 ± 0.0113 (0.97 %)	0.132 ± 0.0096 (1.38 %)	0.041 ± 0.0019 (0.92 %)	0.124 ± 0.0213 (1.22 %)
C20:4 (n-6)	0.048 ± 0.0025 (1.14 %)	0.076 ± 0.0009 (1.02 %)	0.046 ± 0.0044 (0.56 %)	0.063 ± 0.0045 (0.60 %)	0.046 ± 0.0003 (1.13 %)	0.161 ± 0.0105 (1.04 %)	0.051 ± 0.0054 (1.00 %)	0.069 ± 0.0026 (0.72 %)	0.046 ± 0.0051 (1.14 %)	0.092 ± 0.0111 (0.90 %)
C20:5 (n-3) EPA	0.429 ± 0.0165 (10.27 %)	1.047 ± 0.0176 (14.10 %)	0.302 ± 0.0231 (3.65 %)	0.622 ± 0.0426 (5.94 %)	0.496 ± 0.0137 (12.18 %)	2.131 ± 0.1269 (13.74 %)	0.446 ± 0.0399 (8.76 %)	0.759 ± 0.0073 (7.94 %)	0.455 ± 0.0121 (10.20 %)	1.394 ± 0.1755 (13.68 %)
C22:5 (n-3) DPA	0.038 ± 0.0033 (0.90 %)	0.070 ± 0.0015 (0.94 %)	0.034 ± 0.0027 (0.41 %)	0.049 ± 0.0017 (0.47 %)	0.065 ± 0.0018 (1.60 %)	0.203 ± 0.0133 (1.31 %)	0.053 ± 0.0067 (1.04 %)	0.063 ± 0.0019 (0.66 %)	0.041 ± 0.0008 (0.91 %)	0.100 ± 0.0126 (0.98 %)
C22:6 (n-3) DHA	0.861 ± 0.0222 (20.60 %)	1.838 ± 0.033 (24.76 %)	0.434 ± 0.0424 (5.24 %)	0.728 ± 0.1221 (6.95 %)	1.189 ± 0.0229 (29.16 %)	4.207 ± 0.2243 (27.12 %)	1.265 ± 0.1121 (24.84 %)	1.030 ± 0.0656 (10.76 %)	1.225 ± 0.0253 (27.56 %)	2.904 ± 0.3159 (28.50 %)

**ΣPUFA**	1.491 ± 0.0544^ef^ (35.69 %)	3.251 ± 0.0607^c^ (43.82 %)	1.086 ± 0.0837^f^ (13.13 %)	1.799 ± 0.1566^de^ (17.17 %)	1.898 ± 0.0395^de^ (46.55 %)	7.143 ± 0.4061^a^ (46.05 %)	1.96 ± 0.1896^de^ (38.48 %)	2.267 ± 0.0294^d^ (23.69 %)	1.895 ± 0.0404^de^ (42.61 %)	4.791 ± 0.5576^b^ (47.02 %)

**ΣLC n‐3 PUFA**	1.327 ± 0.0421^d^ (31.78 %)	2.955 ± 0.0521^c^ (39.80 %)	0.770 ± 0.0682^e^ (9.31 %)	1.398 ± 0.1663^d^ (13.35 %)	1.751 ± 0.0384^d^ (42.94 %)	6.541 ± 0.3644^a^ (42.17 %)	1.764 ± 0.1587^d^ (34.63 %)	1.852 ± 0.0564^d^ (19.36 %)	1.721 ± 0.0382^d^ (38.67 %)	4.398 ± 0.5041^b^ (43.16 %)

Spring (2020–04–15), Fall (2020–10–21). Results are shown as mean ± SD (n = 2). Different small letters in each row show a significant difference (p < 0.05, Duncan’s multiple range test).

LC MUFA (long chain MUFA) = C20:1, n-9 + C22:1, n-11; LC n-3 PUFA (long chain n-3 PUFA) = EPA + DPA + DHA.

Table 1 shows that, regardless of the season, PUFA was present at a higher relative content (up to 47 %) compared to SFA (up to 38.1 %) and MUFA (up to 30.3 %) in all cuts except the head. In the present study, the LC n-3 PUFAs DHA (up to 29 % of total FA) and EPA (up to 14 %) were the most prevalent PUFAs regardless of cuts and season (Table 1). However, a small level of DPA was also found (up to 1.6 % of total fatty acids), resulting in total LC n-3 PUFA levels of up to 43.1 %. It should also be noted that among the MUFAs, there was a relatively high content of LC MUFAs (especially C22:1 n-11, cetoleic acid, but also C20:1 n-9, gondoic acid). Fall samples had a higher % of SFAs and PUFAs than spring samples, regardless of cuts. In contrast, the % of MUFAs (in total fatty acids) was higher in spring than in fall (Table 1). Furthermore, fatty acids identified in the head had a higher relative content of SFAs (54 % of total FA) and significantly (p < 0.05) lower relative content of PUFAs (13–17 %) compared with the fatty acids from other cuts. The viscera + belly flap had the highest relative level of PUFAs (up to 46 %) and LC n-3 PUFAs (up to 42 %) among all five cuts, regardless of the season. Therefore, these findings suggest that this cut is a promising raw material for extracting these fatty acids.

### Amino acid profile

The absolute and relative content of amino acids is shown in Table 2. In general, four EAA (Lys, Thr, Val, and Leu) and five non-EAA (Arg, Gly, Ala, Glu, and Asp) were predominant (>4.5 %) in the amino acid profile, regardless of season and cut. Among the EAAs, lysine and leucine showed the highest absolute and relative contents compared to other EAAs (Table 2).

**Table 2:** Amino acid content (mg/100 g wet tissue) and as % of the total amount of amino acids of sorted herring filleting co-products and fillet from spring (April) and fall (October).

Amino acids	Backbone		Head		Viscera + Belly flap		Tail		Fillet		FAO/WHOAdult (Infant) (mg/g Protein)
	Spring	Fall	Spring	Fall	Spring	Fall	Spring	Fall	Spring	Fall	
Lysine*	1121.6 ± 25.7^b^ (8.92 %)	1292 ± 71.3^a^ (9.36 %)	777.9 ± 54.6^ef^ (7.78 %)	744.8 ± 2.5^f^ (7.93 %)	880.3 ± 40.6^ed^ (6.63 %)	937.4 ± 36.5 ^cd^ (8.09 %)	999.8 ± 18.3^c^ (7.94 %)	796 ± 37.6^ef^ (7.21 %)	1264 ± 24.1^a^ (9.11 %)	1308 ± 81.3^a^ (9.97 %)	45(57)
Histidine*	294.4 ± 0.3^c^ (2.34 %)	385.7 ± 7.5^a^ (2.80 %)	209.8 ± 18.4^f^ (2.10 %)	189.2 ± 0.8 ^g^ (2.01 %)	285 ± 2.9 ^cd^ (2.15 %)	274.9 ± 0.7^de^ (2.37 %)	261.7 ± 13.3^e^ (2.08 %)	198.5 ± 2.5 ^fg^ (1.80 %)	342.3 ± 7.1^b^ (2.47 %)	355.6 ± 1.6^b^ (2.71 %)	15(20)
Threonine*	607.6 ± 14.3^d^ (4.84 %)	673.3 ± 30.9^bc^ (4.88 %)	449.7 ± 16.4 ^g^ (4.50 %)	442.2 ± 2.8 ^g^ (4.71 %)	718.1 ± 9.3^a^ (5.41 %)	606.7 ± 7.1^d^ (5.24 %)	565.7 ± 23.84^e^ (4.49 %)	496.8 ± 18.6^f^ (4.50 %)	693.1 ± 1.8^ab^ (4.99 %)	651.4 ± 13.3^c^ (4.96 %)	23(31)
Valine*	687.4 ± 21.2^c^ (5.47 %)	797.4 ± 9^b^ (5.78 %)	517 ± 24.7^e^ (5.17 %)	470.9 ± 6.1^f^ (5.01 %)	847.1 ± 19.3^a^ (6.38 %)	690.2 ± 24.5^c^ (5.96 %)	640.1 ± 14.1^d^ (5.09 %)	542.9 ± 1^e^ (4.92 %)	790 ± 0.9^b^ (5.69 %)	693.7 ± 24.7^c^ (5.29 %)	39(43)
Methionine*	409.2 ± 15.9^bc^ (3.26 %)	428.5 ± 35.9^ab^ (3.11 %)	312.4 ± 4.6^d^ (3.13 %)	286.2 ± 30.4^de^ (3.05 %)	371.5 ± 0.7^c^ (2.80 %)	312.8 ± 14.4^d^ (2.70 %)	390.6 ± 16.7^bc^ (3.10 %)	251.8 ± 7.3^e^ (2.28 %)	472.2 ± 15.6^a^ (3.40 %)	418.3 ± 42.6^bc^ (3.19 %)	16(42)
Isoleucine*	560.4 ± 28.4^b^ (4.46 %)	598.5 ± 85.8^ab^ (4.34 %)	377.9 ± 13.6 ^cd^ (3.78 %)	341.4 ± 1.1^d^ (3.63 %)	610 ± 43^ab^ (4.59 %)	547.1 ± 49.9^b^ (4.72 %)	443.9 ± 26.3^c^ (3.53 %)	356 ± 15.6^d^ (3.23 %)	670.2 ± 6.9^a^ (4.83 %)	581.4 ± 8.3^b^ (4.43 %)	30(32)
Phenylalanine*	496.7 ± 64.4^a^ (3.95 %)	601.3 ± 17.9^a^ (4.36 %)	418.6 ± 36.6^a^ (4.19 %)	413.1 ± 0.8^a^ (4.40 %)	508.5 ± 34.3^a^ (3.83 %)	466.9 ± 48.3^a^ (4.03 %)	498.1 ± 5.8^a^ (3.96 %)	422.5 ± 5.6^a^ (3.83 %)	475.1 ± 216.3^a^ (3.42 %)	587.8 ± 66.1^a^ (4.48 %)	19(72)
Leucine*	1018.6 ± 98.2 ^cd^ (8.11 %)	1132 ± 37^ab^ (8.20 %)	693.3 ± 17.4^e^ (6.94 %)	682.9 ± 42.5^e^ (7.27 %)	1146.8 ± 22.5^ab^ (8.63 %)	935.4 ± 29^d^ (8.07 %)	928 ± 22.3^d^ (7.37 %)	716.2 ± 12.3^e^ (6.49 %)	1210.7 ± 10.7^a^ (8.72 %)	1088.7 ± 3.9^bc^ (8.29 %)	59(66)

**Total EAA**	5195.9 ± 226^c^ (41.34 %)	5908.7 ± 169.6^a^ (42.83 %)	3756.4 ± 159.1^e^ (37.59 %)	3570.8 ± 80.4^e^ (38.00 %)	5367.3 ± 46.5^bc^ (40.41 %)	4771.5 ± 3.6^d^ (41.17 %)	4727.9 ± 140.5^d^ (37.56 %)	3780.8 ± 62.6^e^ (34.27 %)	5917.5 ± 244.3^a^ (42.64 %)	5684.8 ± 172.7^ab^ (43.31 %)	
Arginine	630.6 ± 8.3^d^ (5.02 %)	682.9 ± 4.9^f^ (4.95 %)	530.5 ± 28.5^e^ (5.31 %)	438.6 ± 28.2^f^ (4.67 %)	1397.8 ± 6.8^a^ (10.52 %)	998.4 ± 33.3^b^ (8.62 %)	670.9 ± 17.8 ^cd^ (5.33 %)	561.7 ± 20.7^e^ (5.09 %)	714.1 ± 10.1^c^ (5.15 %)	623.6 ± 20.7^d^ (4.75 %)	
Glycine	897.4 ± 3.2^d^ (7.14 %)	925.7 ± 25.6^d^ (6.71 %)	1124.3 ± 44.4^c^ (11.25 %)	1054.6 ± 95.5^c^ (11.22 %)	767 ± 40.5^e^ (5.77 %)	784.2 ± 2.5^e^ (6.77 %)	1359.9 ± 0.5^b^ (10.80 %)	1500.8 ± 16.3^a^ (13.60 %)	856.1 ± 36.5^de^ (6.17 %)	776.5 ± 28.3^e^ (5.92 %)	
Cystine	41.9 ± 3.6^c^ (0.33 %)	52.7 ± 1.4^b^ (0.38 %)	35.8 ± 0.6 ^cd^ (0.36 %)	34.3 ± 1.4 ^cd^ (0.37 %)	69 ± 5.5^a^ (0.52 %)	43.3 ± 3.1^c^ (0.37 %)	37 ± 2.2 ^cd^ (0.29 %)	31.7 ± 0.4^d^ (0.29 %)	61.7 ± 1.0^ab^ (0.44 %)	58.1 ± 9.7^b^ (0.44 %)	
Serine	560.5 ± 13.1^e^ (4.46 %)	616.7 ± 15.7^b^ (4.47 %)	487.8 ± 5.8^f^ (4.88 %)	465.3 ± 3.9^f^ (4.95 %)	699.4 ± 1.1^a^ (5.27 %)	590.4 ± 16.6 ^cd^ (5.09 %)	578.9 ± 9.6^cde^ (4.60 %)	566.6 ± 13.3^de^ (5.13 %)	604.6 ± 1.4^bc^ (4.36 %)	574.4 ± 15.4^de^ (4.38 %)	
Alanine	863.5 ± 8.5^b^ (6.87 %)	947.5 ± 5.2^a^ (6.87 %)	728.7 ± 17.3^d^ (7.29 %)	697.8 ± 40.7^d^ (7.43 %)	950.8 ± 2.7^a^ (7.16 %)	819.3 ± 19.3^c^ (7.07 %)	911.9 ± 3.8^a^ (7.25 %)	916.3 ± 1.2^a^ (8.30 %)	942.1 ± 18^a^ (6.79 %)	869.5 ± 6.7^b^ (6.63 %)	
Glutamic acid	1955.2 ± 122.4^b^ (15.56 %)	2015.8 ± 76.3^ab^ (14.61 %)	1331.4 ± 53.3^e^ (13.33 %)	1273.1 ± 24.2^e^ (13.55 %)	1560.3 ± 14.2^d^ (11.75 %)	1481.9 ± 10.8^d^ (12.79 %)	1775.1 ± 72.9^c^ (14.10 %)	1495.3 ± 45^d^ (13.55 %)	2144.4 ± 18.5^a^ (15.45 %)	2013 ± 33.7^ab^ (15.34 %)	
Aspartic acid	1337.8 ± 84.5^b^ (10.65 %)	1483.1 ± 52.9^a^ (10.75 %)	1002.4 ± 55.6^ef^ (10.03 %)	915.9 ± 31.8^f^ (9.75 %)	1135.9 ± 1.5 ^cd^ (8.55 %)	1046.3 ± 12.2^de^ (9.03 %)	1176.8 ± 19.8^c^ (9.35 %)	1027.9 ± 20.9^e^ (9.32 %)	1476.3 ± 51.1^a^ (10.64 %)	1437.8 ± 47.3^a^ (10.95 %)	
Proline	547.9 ± 15.2^e^ (4.36 %)	592.9 ± 41.5 ^cd^ (4.30 %)	619.6 ± 11.9^c^ (6.20 %)	594.3 ± 31 ^cd^ (6.32 %)	749.8 ± 23.6^ab^ (5.64 %)	591.5 ± 16.2 ^cd^ (5.10 %)	735.3 ± 5.8^b^ (5.84 %)	795 ± 48^a^ (7.20 %)	552.4 ± 5.1^de^ (3.98 %)	517.5 ± 17^e^ (3.94 %)	
Tyrosine	536.4 ± 27.1^b^ (4.27 %)	571 ± 60.2^ab^ (4.14 %)	375 ± 16.7^d^ (3.75 %)	352.3 ± 4.9^d^ (3.75 %)	586.2 ± 11.5^ab^ (4.41 %)	462.3 ± 17.8^c^ (3.99 %)	455.4 ± 3.5^c^ (3.62 %)	357.5 ± 6^d^ (3.24 %)	607.8 ± 10.6^a^ (4.38 %)	569.7 ± 4.9^ab^ (4.34 %)	

Spring (2020–04–15), Fall (2020–10–21). Results are shown as mean ± SD (n = 2). Different small letters in each row show a significant difference (p < 0.05, Duncan’s multiple range test). * Shows essential amino acids (EEA).

### Minerals

Table 3 shows the content of 15 minerals in different cuts during the two seasons. Calcium, sodium, and potassium were predominant among all measured minerals, regardless of cut and season. The tail contained the highest concentration of calcium, followed by head > backbone > viscera + belly flap > fillet. For head, tail, and backbone, spring samples were significantly lower (p < 0.05) in calcium than the samples from the fall season. The sodium content ranged between 147 and 191 mg/100 g ww in all spring cuts except in the head. However, the head had a significantly (p < 0.05) higher sodium content than all other investigated cuts in both spring and fall. Magnesium also showed a similar trend to sodium, with the head containing the highest magnesium concentrations compared to other cuts, with October cuts having a higher magnesium level than those from April (4.6 vs 3.1 mg/100 in the head). Table 3 shows that in both spring and fall, the head had the highest level of heme–iron (up to 2.2 mg/100 g ww), followed by viscera + belly flap ≈ backbone > fillet ≈ tail. The total iron content showed a similar pattern to that of heme–iron, reaching up to 6.3 mg/100 g ww in the head fraction.

**Table 3:** Mineral contents (mg or µg/100 g wet tissue) of sorted herring filleting co-products and fillet from spring (April) and fall (October).

Cont./100 g	Unit	Backbone		Head		Viscera + Belly flap		Tail		Fillet	
		Spring	Fall	Spring	Fall	Spring	Fall	Spring	Fall	Spring	Fall
[Na]	mg	177.94 ± 11.2 ^g^	321.94 ± 4.63^f^	344.98 ± 4.59^e^	1011.42 ± 13.51^a^	181.84 ± 11.29 ^g^	555.4 ± 1.75^c^	190.92 ± 4.01 ^g^	802.09 ± 2.28^b^	147 ± 2.11 ^h^	481.04 ± 5.31^d^
[K]	mg	484.79 ± 3.1^b^	482.49 ± 6.95^b^	255.57 ± 3.4 ^g^	180.17 ± 0.8 ^h^	269.29 ± 0.16^f^	278.51 ± 3.35^d^	255.47 ± 5.37 ^g^	115.09 ± 0.57^i^	646.08 ± 0.53^a^	369.27 ± 3.28^c^
[Ca]	mg	720.97 ± 4.61^e^	1180.81 ± 1.09^d^	2335.18 ± 52.37^b^	2266.35 ± 13.83^c^	135.34 ± 0.08 ^g^	213.79 ± 3.21^f^	2381.99 ± 50.04^b^	3309.66 ± 27.46^a^	64.88 ± 1.9 ^h^	33.14 ± 0.29 ^h^
[Mg]	mg	2.27 ± 0.01 ^cd^	3.01 ± 0.04^b^	3.11 ± 0.04^b^	4.66 ± 0.06^a^	1.292 ± 0.001^e^	2.18 ± 0.01^d^	2.97 ± 0.16^b^	4.62 ± 0.06^a^	2.348 ± 0.002^c^	2.30 ± 0.02 ^cd^
[Fe]	mg	1.35 ± 0.01^e^	1.333 ± 0.003^e^	6.3 ± 0.08^a^	2.935 ± 0.062^b^	1.95 ± 0.03^c^	1.70 ± 0.14^d^	0.71 ± 0.04^f^	0.585 ± 0.005 ^fg^	0.693 ± 0.001^f^	0.462 ± 0.004 ^g^
[Heme-Fe]	mg	0.96 ± 0.21^b^	0.9 ± 0.12b^c^	2.24 ± 0.08^a^	2.14 ± 0.03^a^	0.96 ± 0.05^c^	0.94 ± 0.09^c^	0.27 ± 0.004^d^	0.11 ± 0.02^e^	0.32 ± 0.03^d^	0.12 ± 0.01^e^
[Zn]	mg	0.691 ± 0.004 ^g^	0.57 ± 0.034 ^h^	3.719 ± 0.015^b^	3.193 ± 0.043^c^	0.993 ± 0.001^f^	1.116 ± 0.004^e^	3.661 ± 0.132^b^	5.051 ± 0.042^a^	1.896 ± 0.002^d^	1.006 ± 0.009^f^
[Cu]	mg	0.026 ± 0.002 ^cd^	0.023 ± 0.002^d^	0.024 ± 0.001^d^	0.038 ± 0.004^c^	0.053 ± 0.001^b^	0.068 ± 0.008^a^	0.051 ± 0.012^b^	0.037 ± 0.002^c^	0.079 ± 0.001^a^	0.07 ± 0.003^a^
[I]	µg	42.98 ± 1.40 ^cd^	21.81 ± 5.86^def^	72.65 ± 2.32^ab^	38.72 ± 1.04^cde^	54.33 ± 7.47^bc^	43.87 ± 8.73 ^cd^	91.76 ± 32.91^a^	12.6 ± 0.89^ef^	47.739 ± 1.897^bcd^	8.64 ± 0.61^f^
[Se]	µg	45.36 ± 1.33^c^	46.17 ± 4.43^c^	74.32 ± 5.19^ab^	66.55 ± 10.61^b^	87.17 ± 0.05^a^	81.51 ± 5.27^a^	40.95 ± 14.09 ^cd^	28.94 ± 0.68^d^	36.76 ± 0.42 ^cd^	27.79 ± 1.24^d^
[As]	µg	148.97 ± 0.95 ^cd^	141.12 ± 5.01 ^cd^	124.17 ± 9.63^de^	93.65 ± 10.48^e^	256 ± 3.81^a^	185 ± 13.69^bc^	153.32 ± 50.38 ^cd^	82.63 ± 0.69^e^	214.55 ± 3.48^b^	161.85 ± 17.11 ^cd^
[Cr]	µg	73.11 ± 100.96	7.13 ± 2.17	37.42 ± 45.66	7.24 ± 0.1	3.17 ± 0.58	153.93 ± 205.2	12.79 ± 3.45	8.44 ± 0.54	5.91 ± 0.26	5.84 ± 0.05

[Hg]	µg	4.12 ± 0.03	ND	ND	ND	ND	ND	3.57 ± 1.79	ND	5.2 ± 0.1	0.96 ± 1.35
[Pb]	µg	ND	ND	1.95 ± 0.08	0.83 ± 1.18	ND	ND	ND	ND	ND	ND
[Cd]	µg	2.48 ± 0.03	2.75 ± 0.08	2.16 ± 0.21	3.63 ± 0.37	2.48 ± 0.11	4.59 ± 0.49	ND	ND	ND	ND

Spring (2020–04–15), Fall (2020–10–21). ND stands for Not Detected. Results are shown as mean ± SD (n = 2). Different small letters in each row show a significant difference (p < 0.05, Duncan’s multiple range test).

Table 3 shows that the iodine concentration of all co-product cuts ranged from 12.6 to 91.8 μg/100 g ww, with tail and backbone from fall having the lowest values of 12.6 and 21.8 μg/100 g ww, respectively. April cuts generally had a higher iodine level than cuts from October, and the highest level of iodine was found in the tails. Table 3 shows that the range of selenium content was 27–87 μg/100 g ww, and the viscera + belly flap had the highest level of selenium (87 μg/100 g ww), followed by head > backbone ≈ fillet ≈ tail. However, no significant differences (p > 0.05) between April and October were reported. Table 3 shows that the contents of the three analyzed heavy metals were 0–5.2 μg/100 g ww for mercy, 0–1.95 μg/100 g ww for lead, and 0–4.59 μg/100 g ww for cadmium, with the highest levels found in the backbone, head, and viscera + belly flap, respectively.

### Vitamins E, D, and B12

**Fig. 3:**
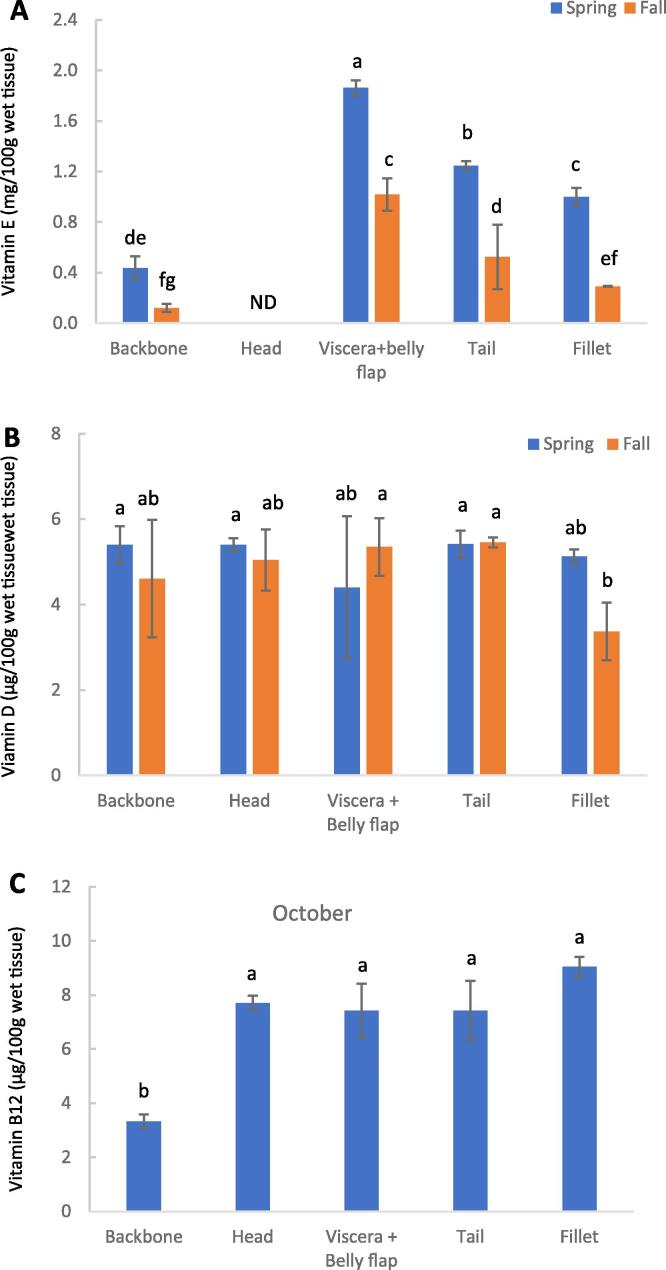
Content of vitamin E (A), vitamin D (B), and vitamin B12 (C) on wet tissue basis of sorted herring filleting cuts from spring (April, only vitamins E ad D) and fall (October, all three vitamins). Results are shown as mean ± SD (n = 2). ND: not detectable. Different small letters show significant differences (p ≤ 0.05).

Among the four tocopherol isomers monitored in this study, only the α-isomer was detected. Fig. 3A shows that all spring samples, except the head, had significantly (p < 0.05) higher levels of α-tocopherol than samples from fall. The Figure also shows that tocopherol was not detected in the head, possibly due to rapid post-mortem consumption and lipid oxidation development (Wu et al., 2022). Among the other cuts, viscera + belly flap contained the highest level of tocopherol, followed by tail, fillet, and backbone, which was observed in both seasons. Furthermore, Fig. 3B shows that the vitamin D content among all cuts ranged between 3.7 and 5.4 μg/100 g ww, but no significant differences between cuts or between spring and fall were observed. However, the fall fillet had significantly (p < 0.05) lower vitamin D than other samples.

Regarding vitamin B12 content, Fig. 3C shows that the head, viscera + belly flap, tail, and fillet had similar amounts of vitamin B12 (7.4–9.0 μg/100 g ww). In contrast, the backbones had significantly (p < 0.05) lower levels (3.3 μg/100 g ww).

## Discussion

### Nutritional and chemical composition of different herring cuts

The head showed the highest lipid content among the five herring cuts in April samples (Fig. 2B) partly because the brain contains a substantial amount of lipids. For example, Hong et al. (2014) found that fish brains contained 48–65 % of lipids (ww basis), depending on species, habitat, and food consumed. Our data agreed with those of Ahmmed et al. (2021), which reported that in blue mackerel (*Scomber australasicus*), the head had the highest lipid content (12.3 % ww) compared to other co-products cuts, such as white muscle (1.3 % ww), liver (9.4 % ww), and viscera (8.1 % ww). Our findings of the head and tail containing lower protein and higher ash compared to other cuts (Fig. 2C and D) align with the fact that the tail and head have higher bone content. Conversely, the intestines + belly flap are nearly deficient in bones yielding less ash. Overall, the proximate compositional data indicated that all herring co-product cuts could be valuable protein and lipid sources. In many cases, these macronutrients were present at levels comparable to, or higher, than in the fillet, providing strong incentives for their added value to minces or ingredients. Furthermore, bony parts enriched in ash could be excellent sources for recovering minerals such as calcium (Kim & Jung, 2007).

The seven fatty acids which dominated the identified fatty acids in all cuts (C14:0, C16:0, C16:1, C18:1, C22:1, C20:5, and C22:6) agreed with the study of Bazarnova et al. (2020) which comprised Atlantic (*Clupea harengus*) and Pacific (*Clupea pallasii*) herring fillets. Our findings are also in agreement with fatty acid data of Atlantic herring (*Clupea harengus*) provided by the US Department of Agriculture (USDA) Agricultural Research Service (USDA, 2019). Our findings of relatively higher PUFA levels compared to SFA and MUFA in all cuts, except the head, were also consistent with previous studies. Notably, these previous studies showed that the PUFAs of herring fillet mince was approximately 41 % (Larsson, Almgren, & Undeland, 2007) and 48 % (Szlinder‐Richert, Usydus, Wyszynski, & Adamczyk, 2010) of total fatty acids, which was significantly (p < 0.05) higher than SFAs and MUFAs. Our findings of SFAs being prevalent within the head fraction concurs with Abiona et al. (2021). Specifically, they reported that the relative content of SFAs in gills and heads of catfish (*Claris macrocephalus*) and Titus fish (*Scomber scombrus*) were 40–45 % of total fatty acids. Furthermore, our findings of DHA and EPA being prevalent in the pool of PUFAs agrees with the data of Keinänen et al. (2017), which studied whole-body homogenates of Baltic herring (*Clupea harengus*). The bioactivity of the LC n-3 PUFA is well reviewed and described (Calder, 2015), translating into roles such as preventing inflammatory and cardiovascular diseases as well as supporting optimal development and functioning of the human nervous system, brain, and vision (Tocher et al., 2019). Few animal studies have also reported that LC n-3 PUFA may prevent life-style related diseases, such as type 2 diabetes, metabolic syndrome, and atherosclerosis (Tsutsumi et al., 2021). It is also interesting to note that cetoleic acid (C22:1 n-11) may improve the efficiency of the in vivo conversion of alfa linoleic acid (ALA) to EPA and DHA (Østbye et al., 2019), as relatively high content of LC MUFAs (e.g. C22:1 n-11 and C20:1 n-9) were observed in all cuts (Table 1),. Thus, our results indicate that herring co-products are excellent sources of both LC MUFA and LC n-3 PUFA rich lipids. Based on the data in Table 1, all cuts exceeded EPA/DHA-levels required to support functional health claims related to normal brain function and vision (≥40 mg DHA/100 g ww) and normal heart function (≥ 40 mg EPA + DHA/100 g ww) (European Commission, 2006). Furthermore, fall viscera + belly flap also exceeded levels needed for claims on normal blood triglyceride levels (≥2 g EPA + DHA/100 g ww) and normal blood pressure (3 g DHA/100 g ww) (European Commission, 2006). Therefore, these cuts may provided necessary supplementation of these nutrients it processed and utilized for consumption.

Our findings of all cuts being especially rich in four EAAs (Lys, Thr, Val, and Leu) and five non-EAAs (Arg, Gly, Ala, Glu, and Asp) agree with the amino acid composition data of Atlantic herring (*Clupea harengus*) provided by the USDA Agricultural Research Service (USDA, 2019) and by Bazarnova et al. (2020). Lysine was the predominant EAA in all cuts, which is highly relevant since it is the limiting amino acid in cereals, especially wheat products (Meybodi, Mirmoghtadaie, Sheidaei, & Mortazavian, 2019). Thus, herring products derived from co-products could be promising food sources to satisfy the human requirement for lysine. Based on the EAA requirements for humans as reported by the World Health Organization (WHO, 2007) (Table 2), a meal consisting of 100 g co-products, cooked as is, would cover the EAA requirements set by WHO for adults regardless of cut. However, valine, phenylalanine, and methionine did not meet the requirements set by WHO for infants. Therefore, these findings indicate that herring co-products could provide enough EAAs for adults, but other protein sources should be supplemented in meals for infants.

The order of calcium found in the co-products was identified as tail > head backbone > viscera + belly flap > fillet (Table 3), revealing that bones were the primary calcium source. Similar findings were also reported by Kim et al. (2007), who revealed that around 60 % of the inorganic minerals of fishbone were calcium. From a nutritional point of view, calcium is essential for numerous functions in the body, including strengthening teeth and bones, nerve function, and many enzymatic reactions that require calcium as a cofactor (Kim & Jung, 2007). All cuts exceeded the levels approved by EFSA to make functional health claims based on calcium (≥120 mg/100 g ww) (European Commission, 2006). However, it is important to stress that levels will be significantly reduced if the bones are removed from the fish.

The sodium levels identified generally agreed with the data reported by Swedish National Food Agency (SLV, 2021). The head containing the highest sodium levels among the five cuts (Table 3) may partly be due to the gills absorbing sodium when submerged in refrigerated seawater (RSW) before filleting. At the same time, the skin barrier likely protected the other tissues. This hypothesis is supported by Hwang, Lee, and Lin (2011), who reported that the gills are the primary organs conducting the internal ionic and acid-base regulation, which may allow salt ions from seawater to diffuse easily into the gills after death. From a nutritional point of view, sodium contributes to conducting nerve impulses, contracting and relaxing muscles, and maintaining a proper balance of water and minerals. However, the intake should not exceed 6 g per day. Besides affecting the nutritional properties of the co-products, salt (NaCl) absorbed by the herring tissues could also be a potential pro-oxidant, which may promote the development of lipid oxidation (Mariutti & Bragagnolo, 2017). As for magnesium levels, heads had the highest concentrations of magnesium, which is relevant since magnesium is a cofactor in more than 300 enzyme systems that regulate diverse biochemical reactions in the body.

Interestingly, iron was also mainly enriched in the heads, and the levels found (2.9–6.3 mg/100 g ww) well-exceeded levels needed to make functional health claims based on Fe (≥2.1 mg/100 g ww) (European Commission, 2006). The European Commission (2021) recently reported iron as one of the most critical trace minerals within human nutrition based on its involvement in oxygen transport via the production of red blood cells/hemoglobin (Hb). It has been further recognized for its roles in cognitive function, energy-yielding metabolism, and the immune system. However, the form of iron in the diet is crucial for its bioavailability. Moustarah and Mohiuddin (2021) reported that about 25–35 % of dietary heme iron gets absorbed, while only 2–17 % of dietary nonheme iron is absorbed. Thus, the fact that the heme–iron of the five herring cuts contributed to between 36 and 73 % of the total iron is fundamental from a nutritional point of view. Further, studies show that the red blood cell (RBC) membrane can maintain heme soluble during gastrointestinal digestion, facilitating its uptake. Earlier findings that Hb of the RBCs is the main contributor to heme–iron in fish (Wu, Richards, & Undeland, 2022) are also nutritionally relevant. Given that heme–iron is mainly in the form of Hb, it can be distributed across the different cuts depending on the distribution of blood vessels in those tissues. Brill and Bushnell (2006) reported that the main blood vessels in most cold-blooded fish run along the backbone and radiate outwards to the small vessels that supply oxygen to visceral organs and muscles. Furthermore, fish acquire oxygen from the water through the gills, likely explaining why the head contains more blood vessels than other anatomical parts. In our previous study, herring backbones showed higher total iron and Hb than cod and salmon backbones (Wu, Abdollahi, & Undeland, 2021), pointing to a higher degree of vascularization in small pelagic species than in white fish and salmonoids. It is also important to note that active bleeding is not applied to small pelagic fish species like herring and mackerel, which is why most blood remains in the tissue post-mortem. However, small amounts leak into RSW-waters (Osman et al., 2015). Overall, these findings suggest that herring co-products and refined products thereof may serve as important sources of highly bioavailable iron for humans. Notably, it is also important to highlight the ongoing dietary protein shift since a change to a fully vegan diet often yields low iron deposits and mild to severe anemia, not least in women of fertile ages (Pawlak, Berger, & Hines, 2018).

Iodine and selenium are two distinguished minerals in seafood compared to other animal foods (e.g., mammals and poultry), which are deficient in these minerals (Aakre et al., 2019). The present study revealed that herring is not an exception. The iodine concentration of all co-product cuts, except the tail and backbone from fall, were higher than the 22.5 µg/100 g ww, which is needed to claim that food is a dietary source of iodine as authorized by the European Commission (2021) (Table 3). Iodine is an essential component of the thyroid hormones thyroxine and triiodothyronine, which are necessary for protein synthesis and enzymatic activity, and are critical determinants of metabolic activity (McManus & Newton, 2011). Likewise, selenium plays vital roles in many metabolic responses, such as reproduction, DNA synthesis, and protection from oxidative damage and infection (Aakre et al., 2019). Notably, levels of selenium in all herring cuts exceeded those needed to make functional health claims according to EFSA (8.25 µg Se/100 g ww) (European Commission, 2006). Our data thus indicate that herring co-products have the potential to become functional foods due to their significant levels of iodine- and selenium.

Metals such as lead, cadmium, and mercury accumulate in the marine food web at levels that, in some areas, can be toxic to aquatic organisms and pose a health risk for humans who consume them (Polak-Juszczak, 2009). For example, lead and mercury have been reported to cause congenital disabilities and affect the neurological system. Likewise, cadmium was reported to cause lung cancer and affect human fertility and reproduction (Boalt, Miller, & Dahlgren, 2014). The European Commission (2015) designated the maximum levels for specific contaminants in foodstuffs to be 50 μg/100 g ww for mercy, 30 μg/100 g ww for lead, and 5 μg/100 g ww for cadmium. Our results revealed that the content of all three heavy metals in the herring co-product cuts (Table 3) is lower than these maximum levels, far below in the case of mercury and lead. Therefore, this indicates that herring co-products do not pose any potential risks to human health regarding heavy metal levels and exposure.

Vitamin E (α-tocopherol) is a highly efficient membrane-bound antioxidant important for the skin, nervous system, heart, and circulatory system (McManus & Newton, 2011). The viscera + belly flap containing the highest level of tocopherol among the five cuts (Fig. 3A) may partly be related to the liver being the primary organ that accumulates α-tocopherol to maintain the α-tocopherol status of other organs and tissues (Hamre, 2011). Notably, fatty fish like herring contain high lipid-soluble vitamin D levels (Abdollahi, Wu, & Undeland, 2021). The European Commission (2021) reported that vitamin D could aid calcium absorption, play a role in cardiovascular function, and support healthy inflammatory responses. In Nordic countries, vitamin D is one of the few vitamins where deficiencies can be seen within the population due to the year's long dark season (Trollfors, 2022). The observed vitamin D content of herring fillets (3.7 and 5.1 μg/100 g ww in fall and spring, respectively) agreed with data reported by the USDA (2019), releaving 4.2 μg vitamin D/100 g ww Atlantic herring (*Clupea harengus*) fillet. The level found in backbones (Fig. 3B) was higher than in salmon and cod backbones or mechanically separated muscle from these sources (Abdollahi, Wu, & Undeland, 2021). Vitamin d-levels exceeded those needed to make a functional health claim; 0.75 µg vitamin D/100 g (EFSA) (EC Commission, 2015), suggesting that herring co-products are excellent sources for the production of vitamin d-rich products.

Vitamin B12 is well recognized as being necessary for DNA synthesis, red blood cell formation, and neurological function (Ozogul et al., 2021). Deficiency of this vitamin can be associated with megaloblastic anemia, neurological disorders, myelopathy, and memory impairment (McManus & Newton, 2011). Our data revealed that all four co-product cuts showed similar amounts of vitamin B12 as the fillets, and the identified values were identical to those reported by the Swedish National Food Agency (SLV, 2021) for herring fillets (8.79 μg/100 g ww). Although the backbones had the lowest concentrations of vitamin B12 (3.3 μg/100 g ww) of all investigated cuts, they still exceeded the level approved by the European Commission (2021) to claim that a food item is a high vitamin B12 source; > 0.75 μg/100 g ww. Given the current need among many consumers to switch to diets containing less red meat, new sustainable vitamin B12 sources have become highly important. Furthermore, this may be an added source to supplement this vitamin.

### Variations between typical catches from the spring and fall season

That the lipid content of fall samples was higher than in samples from spring (Fig. 2B) agreed with our earlier findings (Larsson, Almgren, and Undeland (2007). In our previous study, we reported that de-skinned herring fillets from fall showed a significantly (p < 0.05) higher total lipid content than similar samples from spring (7.5 % vs 1.5 % ww). These significant differences (p < 0.05) between spring and fall could be attributed to spawning and feeding patterns. Frantzen, Måge, Iversen, and Julshamn (2011) investigated the effect of season on the physical and biological parameters of herring (*Clupea harengus*) from the Norwegian Sea. They reported that the lipid content of herring fillets sampled at the end of the feeding season (October) was 17 g/100 g ww. In contrast, herring caught in spring (April), after a long period of starvation followed by spawning, showed the lowest lipid contents of the year, with an average of 4.0 g/100 g ww. That protein contents of head, fillet, and backbone were similar between spring and fall (Fig. 2C) agrees with Slotte (1999), who reported that the proteins remained relatively constant in Norwegian spring-spawning herring caught both spring and fall. These findings could be partially explained by the fact that proteins play multiple physiological roles in fish, from muscle contraction to oxygen transport and various enzymatic actions (Andersen, Waagbø, & Espe, 2016). The spring samples of viscera + belly flap and tail still had higher protein levels than corresponding fall samples (Fig. 2C) due to higher levels of roe and milt, both high in protein, in viscera from spring than fall (Ahmmed et al., 2021). Moreover, spawning migration in the spring could require more muscle mass in the tail section compared to the fall, partly explaining these differences in protein. That all cuts (except viscera + belly flap) contained higher ash levels in fall compared to spring samples (Fig. 2D) was most likely related to the longer post-mortem storage of the herring in RSW-tanks in October compared to April (72 h vs 6 h). Furthermore, extended RSW-storage allows to for more salt to migrate to the tissue via osmotic pressure (Supplementary material, Table S1).

The fall samples showed a higher % of SFAs and PUFAs, and a lower % of MUFAs than the spring samples, which agreed with Szlinder-Richert et al. (2010). These authors reported a higher % of PUFAs and a lower % of MUFAs in October-caught herring (*Clupea harengus*) lipids than in April-caught fish lipids. Similarly, Hamre, Lie, and Sandnes (2003) reported that the lipids of herring fillets from October had a higher % of SFAs and PUFAs and a lower % of MUFAs compared to lipids from March fillets. This change in the fatty acid composition may be due to the preferred use of PUFAs and SFAs for catabolism and spawning, while the MUFAs are spared in these processes. It is reported that SFAs serve as energy supplies (Szlinder-Richert et al., 2010), which is why it is logical that their concentrations increase during the intense feeding period in the fall. That the April viscera + belly flap (including roe) lipids showed a higher % of DHA than in October (Table 1) could be attributed to the fact that DHA accumulates in the roe during the spawning period (Lu & Takeuchi, 2004).

Our study revealed that all fall cuts contained higher sodium and magnesium than spring cuts (Table 1). Sodium and magnesium are seawater's two most abundant cations (Kim, Ko, Kang, & Han, 2010). As stated earlier (Table S1), herring from October was subjected to much longer post-mortem storage in RSW tanks before filleting (48 vs 24 h). Thus, these results indicate that the time in RSW tanks should be minimized, where possible, to prevent herring tissues (particularly the gills and head) from absorbing substantial levels of salt ions from seawater.

All cuts, except the head, had higher levels of α-tocopherol in spring than in fall samples (Fig. 3A), which agrees with Syväoja et al. (1985). They measured α-tocopherol contents in twelve different marine fish (including herring) caught in spring and fall and found that fish caught in spring had higher tocopherol than fish caught in the fall. The observed seasonal variation may be due to the involvement of tocopherol in the sexual maturation of fish, explaining their peak during the spawning season (Syväoja et al., 1985). Similarly, Bragadóttir, Pálmadóttir, and Kristbergsson (2002) found that the tocopherol content in capelin (*Mallotus villosus*) was very high during the spawning period in the spring but then steadily declined throughout the remainder of the year.

### Potential utilization of herring co-products

The present study provided a comprehensive investigation of the nutritional composition of sorted herring co-products, which could benefit further utilization in the production of both food products and nutraceuticals. Overall, all herring co-product cuts contained high levels of proteins with a balanced level of essential amino acids (Fig. 2A and Table 2). This makes the production of minces, hydrolysates, thermostable protein dispersions, collagen, gelatin, protamine, specific peptides, and amino acids promising (Rustad, Storrø, & Slizyte, 2011). In the case of the backbones, we have earlier reported that large amounts of residual muscle (up to 80–85 %) can be recovered in high yield using mechanical meat/bone separation (Abdollahi, Wu, and Undeland (2021). This mince is very promising for fish burgers, for example. The remaining bone could be used to develop mineral-rich nutraceuticals by simple and low-cost operations involving weak alkali, acid, and hydrogen peroxide solutions, followed by drying and grinding (Rustad, Storrø, & Slizyte, 2011). Although the head also contains large amounts of muscle, the mechanical meat–bone separation technology would create a very complex mince with high susceptibility to oxidation (Wu et al., 2022). Thus, we believe other valorization methods are more appropriate. Auspicious results have been revealed when recovering functional proteins from the head and backbone via the pH shift technology, without and with the addition of extra antioxidant-containing raw materials such as berry and fruit pomace (Abdollahi et al., 2020, Abdollahi and Undeland, 2019, Zhang et al., 2022). Much research has also been carried out on producing protein isolates or hydrolysates from mixed fish co-products (Abdollahi et al., 2021, Ghaly et al., 2013, Sajib et al., 2020), which would be a promising route also for some of the more challenging and complex co-products in a separate form; e.g., the visceras + belly flap and/or the head.

The high lipid content and LC n-3 PUFA and LC MUFA content of the visceras + belly flap also make it particularly promising for oil recovery purposes. However, the ordinary production of fish oil involving heating, pressing, and centrifugation may be stressful to the sensitive marine lipids, leading to rapid degradation and oxidation. Thus, more gentle oil extraction technologies for fish co-products can be advantageous to generate high-quality fish oils. For example, Bruno, Kudre, and Bhaskar (2019) propose that ultrasound-assisted enzymatic extraction is a promising method for improving the oil yield (up to 67 %) from the heads of carp (*Labeo rohita*). Furthermore, this method resulted in higher quality oil with low oxidation status and free fatty acid (FFA) concentration than traditionally produced oil. Similarly, we reported how oil recovery via the pH-shift process generated higher oil quality than classic heat-based separation in terms of lipid oxidation and FFA (Abdollahi & Undeland, 2020).

When it comes to the tail, which had a very high level of collagenous tissue, we see this as an interesting substrate for collagen extraction, potentially after first recovering the small residues of muscle via a meat bone separator. Other direct uses as snacks are also promising, in which smoked salmon fins are already an established product.

Notably, retaining the quality of the herring co-products is one of the critical factors for their successful utilization as consumable products. Our previous study reported a high susceptibility of all five sorted herring cuts to lipid oxidation during their storage on ice (Wu et al., 2022). Therefore, these findings, along with our previous investigations, indicate that herring co-products should be processed immediately after filleting unless antioxidants are added to inhibit lipid oxidation during storage.

## Conclusion

As identified in this study, herring filleting co-products (head, backbone, viscera + belly flap, tail) contribute to a large proportion of the whole herring weight (up to 60 %), as demonstrated from five sampling months between March and October. From thorough investigations of April and October samples, we found that all four investigated co-product cuts contained high levels of proteins with balanced EAAs, lipids rich in LC n-3 PUFAs and LC MUFAs, vitamins E, D, and B12, as well as iodine, selenium, calcium, and heme–iron. In many cases, these macro- and micronutrient levels were higher in the co-product cuts than in the fillet, which today is usually the only cut that goes to food production. Many of the monitored nutrients exceeded levels required by EFSA to make functional health claims, which further reinforces the potential of herring co-products as food or nutritional supplements. Importantly, the co-product samples did not pose any risk to human health based on analyzed mercury, lead, and cadmium levels and are thus suggested to be safe for human consumption. Most macro- and micronutrients significantly varied depending on the catching season, time in RSW tanks, and types of cut, which should be considered when designing their valorization pathways. In this respect, the present study can serve as a valuable basis for tailoring the best combination of value-adding technology and herring co-product cut. More diversified and tailored utilization of all fish parts can provide better profit for fish companies and maximize the retention of valuable nutrients in the food chain.

## Declaration of Competing Interest

The authors declare that they have no known competing financial interests or personal relationships that could have appeared to influence the work reported in this paper.

## Data Availability

Data will be made available on request.
