# Supplementary material for: Five cuts from herring (Clupea harengus): Comparison of nutritional and chemical composition between co-product fractions and fillets
Source: Food Chem X. 2022 Oct 25;16:100488. doi: 10.1016/j.fochx.2022.100488 (PMC9636446; doi:10.1016/j.fochx.2022.100488)

**Supplementary data**

**Table S1.** Information about catching area and pre-process storage time for the herring used in the five sorting trials. All five batches are from 2020.

**Supplementary data**

| Data to record | Mar 24^th^ | April 15^th^ | Aug 26^th^ | Sept 30^th^ | Oct 21^st^ |
| --- | --- | --- | --- | --- | --- |
| Fishing location | Limfjord | Limfjord | North Sea | Kattegat | North sea |
| Time of fishing | 22-Mar | 12 and 14 April | 24-Aug | 29-Sep | 16-Oct |
| Time on board boats | 6h | 6h | 45h | 26h | 72h |
| Time in the factory after landing until filleting | 24h | 24h | 12h | 3h | 48h |

**Table S2.** Method supplementary details

| Analysis | Details |
| --- | --- |
| Crude protein analysis | The crude protein content of dried and powdered cuts was determined according to the Dumas combustion method using a LECO Trumac Nitrogen analyser (LECO Corporation, Michigan, USA). Samples of freeze-dried powder (∼1 g) were combusted at a temperature of 1100 °C within the furnace. Protein values were calculated from the measured total nitrogen % by multiplying by 5.58 (Mariotti et al. 2008). EDTA Calibration Sample (LECO Cooperation) was used as a standard. |
| Methylation for fatty acid analysis using GC-MS | 1. Ten % (v/v) acetylchloride in MeOH is carefully prepared on ice and with full protection. 2. Add 1 mL toluene. 3. Add 1 ml methanol:acetylchloride. 4. Shake (do not vortex). 5. Leave overnight or put in a heat block, 60°C, 120 min. 6. Add 1 mL milliQ water. 7. Add 1.5 mL petroleum ether. 8. Vortex, 10 sec. 9. Centrifuge 2500 g x 5 min. 10. Transfer the upper part to new tubes. 11. Repeat step 7 and pool sample. 12. Evaporate, N_2_, 40˚C, 30 min or longer, until dryness. 13. Dissolve the sample in 500 µL isooctane. 14. Transfer 200 µl to GC vial, with inserts. 15. Run on GC-MS or save in -20 °C freezer until analysis (a few days). |
| Amino acid analysis | Instrumental parameters:  The LC-MS analysis was performed by an Agilent LC 1260 infinity HPLC system equipped with a G1312B 1260 binary pump, an autosampler, and a temperature-controlled column oven. This was coupled to an Agilent 6120 LC-MS detector. Analytical separation was achieved with a thermos Luna C18 2 250 × 4.6 3μ × 2 (Phenomenex) column with a gradient of A: 3% MeOH, 0.2% formic, and 0.01% acetic acid in h20. B: 50/50 MeOH/H2O 0.2% formic and 0.01% acetic acid with a flow rate of 0.6 ml/min. Gradient Time 0 min A 94 B 6, Time 8 min A94 B 6, Time 20 min A80 B20, Time 27 min A80 B20, Time 29 min A94 B 6. Total run time 40 min. Data acquisition was performed using Selective ion monitoring positive APCI using the following parameters: Drying gas (N2) with a flow of 4 L/min, nebulizer pressure of 55 psi, drying gas with a temperature of 320, and a vaporing temperature of 425, capillary voltage of 3000 V, corona current of 4uA and a fragmentor voltage of 55 eV (Hinchcliffe et al. 2019). |
| Mineral analysis | \| Instrument parameters: \|  \| \| --- \| --- \| \| Instrument \| Perkin Elmer DRCe \| \| RF power \| 1200W \| \| Sample and skimmer cones \| Nickel \| \| Pulse stage voltage \| 1250 volts \| \| Lens voltage \| Optimised daily \| \| Analogue voltage \| 1900 volts \| \| Plasma gas (argon) \| 15 l min^-1^ \| \| Nebuliser gas (argon) \| 0.9 l min^-1^ \| \| Sweep readings \| 15 \| |

References

Mariotti, F., Tomé, D., & Mirand, P. P. (2008). Converting nitrogen into protein—beyond 6.25 and Jones' factors. *Critical reviews in food science and nutrition*, *48*(2), 177-184.

Hinchcliffe, J., Carlsson, N. G., Jönsson, E., Sundell, K., & Undeland, I. (2019). Aquafeed ingredient production from herring (*Clupea harengus*) by-products using pH-shift processing: effect from by-product combinations, protein solubilization-pH and centrifugation force. *Animal Feed Science and Technology*, 247, 273-284.

**Figure S1.** Photo documentation of the whole herring used in the five samplings between March to October 2020


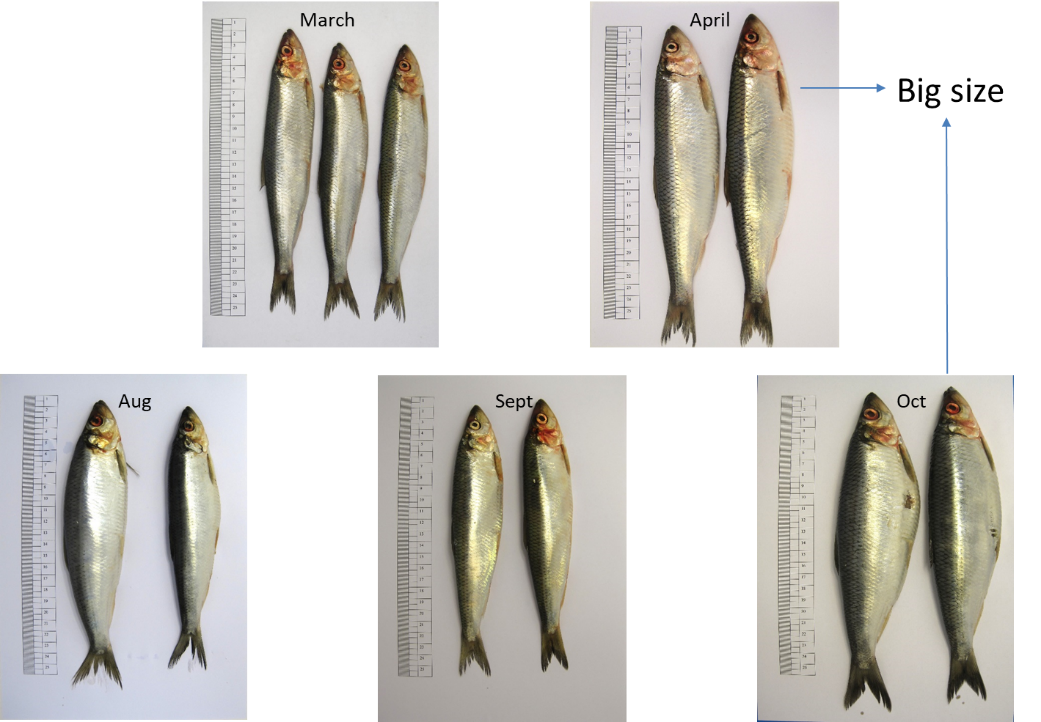


**Figure S2.** Photo documentation of the different cuts emerging from the April and October sampling


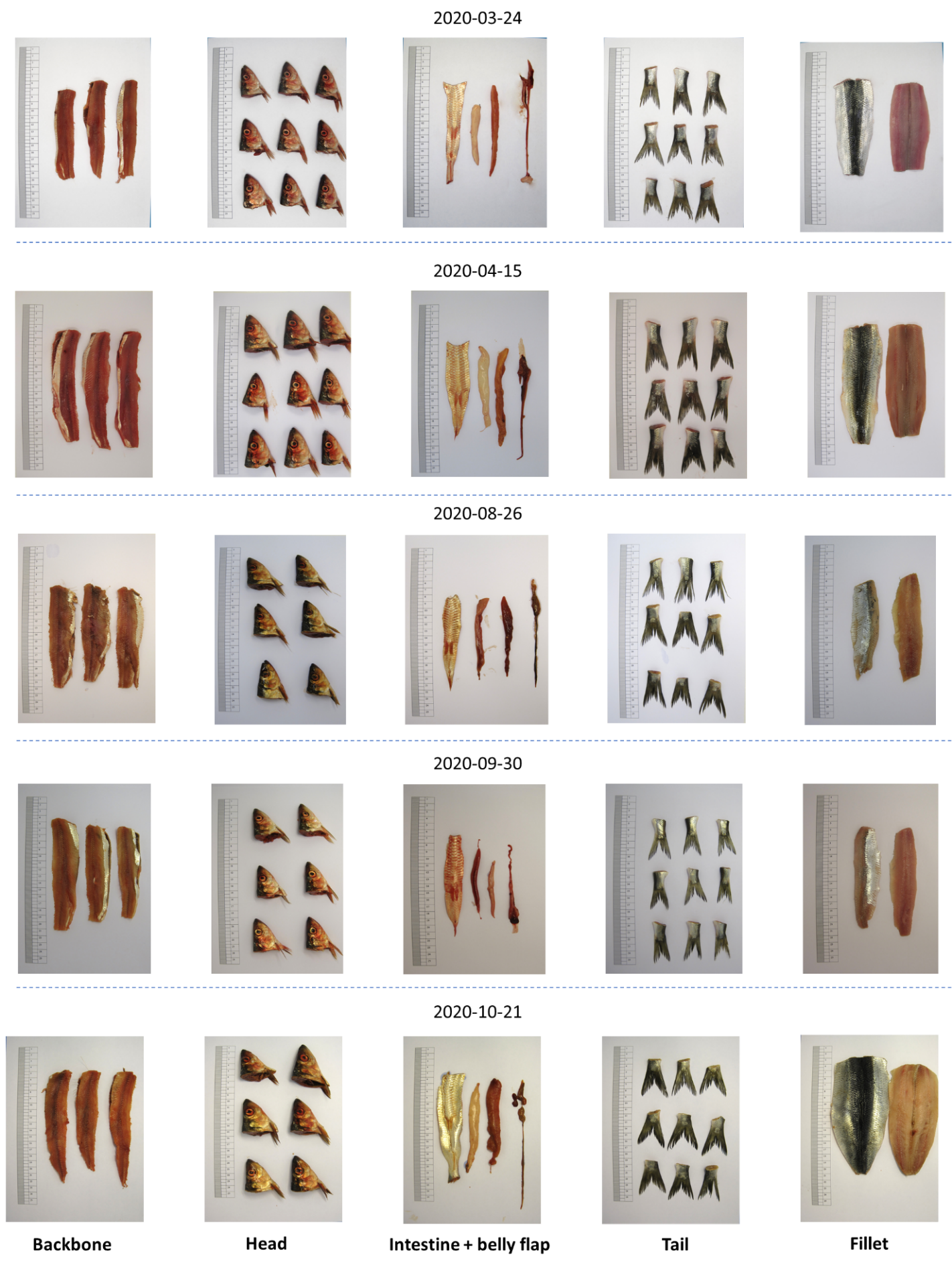


**Figure S3. The amino acid requirements from FAO/WHO**


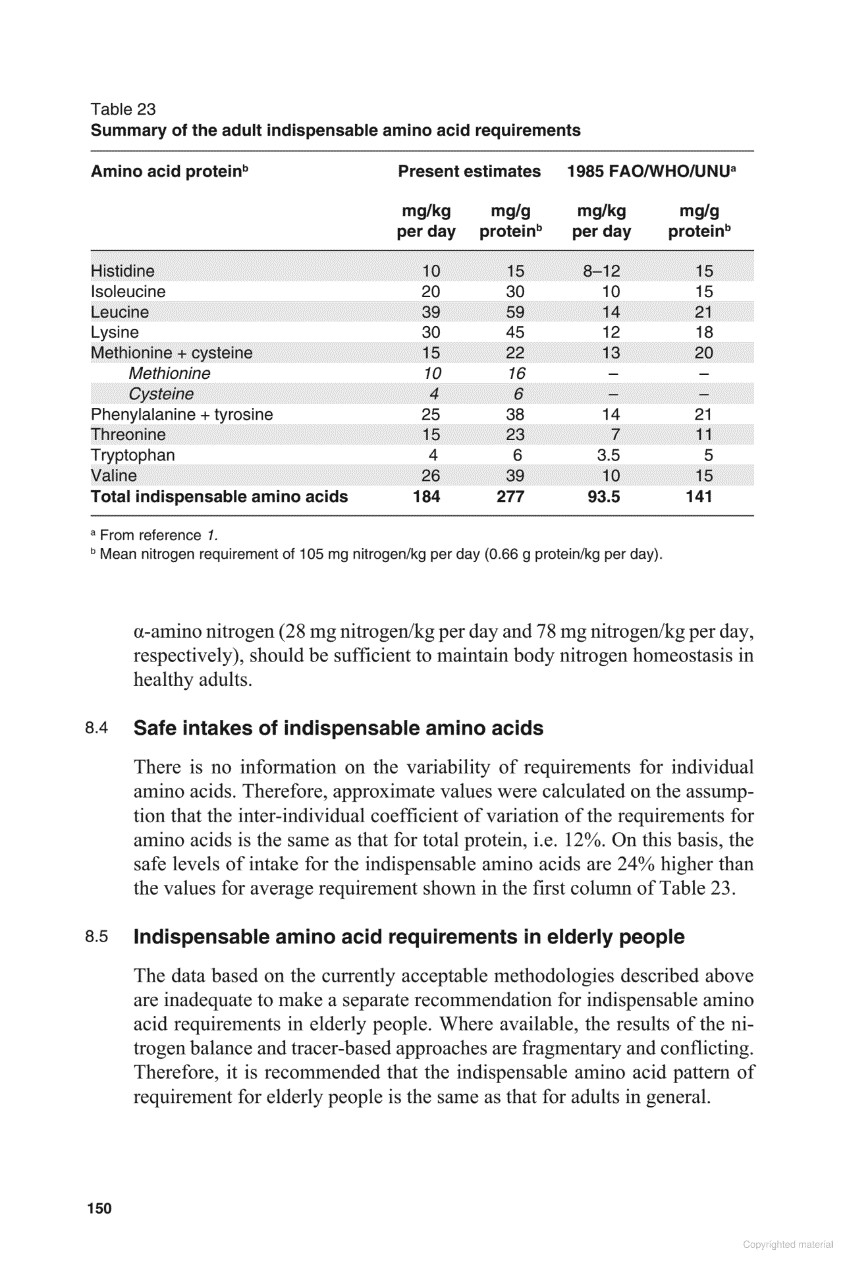

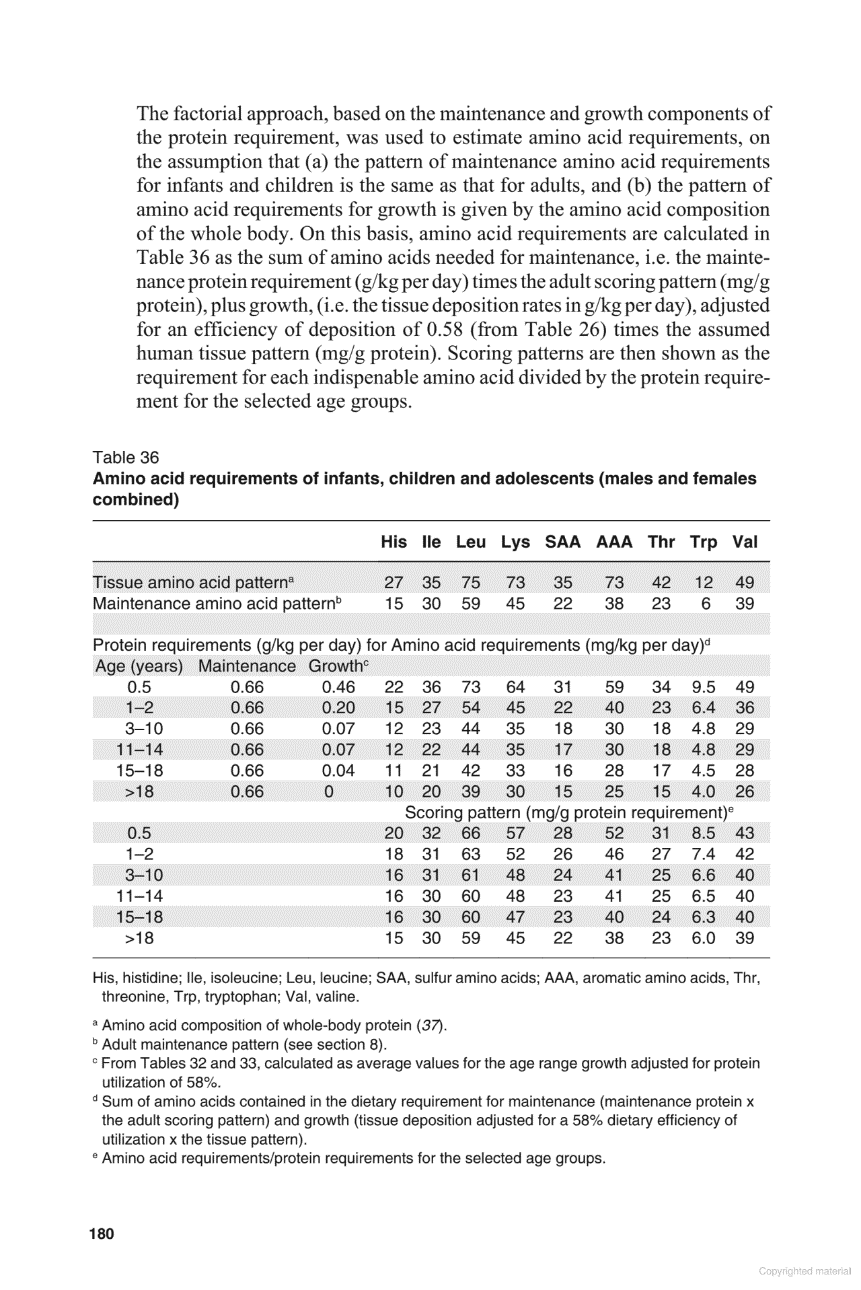

Supplement: Supplementary data 1 [file mmc1.docx]
